# Morphometric and immunohistochemical reorganisation of the palatine tonsil in chronic tonsillitis and its response to combined ozone-ultrasound therapy

**DOI:** 10.3389/fsurg.2026.1878051

**Published:** 2026-08-26

**Authors:** Nigina Alimova, Dilnoza Khasanova, Nigora Dustova, Nigora Asadova

**Affiliations:** Bukhara State Medical Institute named after Abu Ali ibn Sino, Bukhara, Uzbekistan

**Keywords:** bcl-2, CD68, chronic tonsillitis, immunohistochemistry, ki-67, morphometry, ozone therapy, palatine tonsil

## Abstract

**Background:**

Chronic tonsillitis is a common upper respiratory tract disease for which there is no objective tissue-level basis for judging conservative treatment. We characterised the morphometric and immunohistochemical reorganisation of the palatine tonsil in chronic tonsillitis and its response to a combined ozone-ultrasound protocol.

**Methods:**

In a single-centre observational study with a pre-post design and no randomised comparator group, we examined 480 palatine tonsil specimens: histologically normal controls (*n* = 160), chronic tonsillitis (*n* = 180) and post-treatment specimens (*n* = 140). Sections underwent haematoxylin-eosin and Masson's trichrome staining and immunohistochemistry for Ki-67, CD68 and BCL-2 (Ventana BenchMark XT, Roche; 1:100), with immunopositive cells counted in 5–10 high-power fields at x400. The multi-stage ozone-ultrasound protocol (patent FAP20230057) was applied to 180 patients aged 4–40 years assessed before and after treatment.

**Results:**

Chronic tonsillitis showed deepened crypts (+412.3%), capsular thickening (+252.6%), epithelial erosion (+152.5%) and stromal fibrosis (+54.0%), whereas epithelial thickness changed marginally (+6.6%). Follicle density fell by 54.7% and germinal centre number by 22.1%, while germinal centre area was preserved (−4.3%; *p* = 0.09), pointing to loss of follicles rather than involution of individual centres. Ki-67, CD68 and BCL-2 rose in every compartment and fell after therapy (all *p* < 0.05). The clinical severity index dropped from 4.28 to 2.78 points (mean change 1.50; 95% CI 1.17–1.83).

**Conclusion:**

Chronic tonsillitis produces a reproducible pattern of morphometric and immunohistochemical reorganisation that responds in a coordinated way to combined ozone-ultrasound therapy, giving an objective tissue-level framework for judging conservative treatment.

## Introduction

Upper respiratory tract infections remain the leading cause of acute illness worldwide and a common reason for an outpatient visit. The Global Burden of Disease Study 2021 put the figure at 12.8 billion new episodes (95% uncertainty interval 11.4–14.5) in that year alone, with incidence highest in children under two and the largest absolute number of cases between five and nine years (1). Tonsillar inflammation accounts for much of this load. Bacterial tonsillitis underlies 15%–30% of sore-throat episodes in children and 5%–10% in adults (2), and roughly one person in nine reports recurrent tonsillitis at some point in life (3). In otolaryngology clinics the diagnosis is made in about a quarter of patients who present with persistent throat complaints (4). Surgical guidelines have tightened and elective tonsillectomy has declined, yet admissions for tonsillitis rose by 310% between 1991 and 2011 in one national health system (5). Effective conservative treatment, and some objective way of judging whether it works, therefore remain unmet needs.

The palatine tonsils sit at the entrance to the respiratory and digestive tracts and act as inductive sites of mucosal immunity. Their crypt architecture multiplies the surface available for antigen sampling and, under normal conditions, drives reactive hyperplasia of lymphoid follicles with active germinal centres (6, 7). Persistent microbial colonisation upsets that balance. Polymicrobial biofilms anchored inside the crypts (8) disturb local immune homeostasis, and a self-sustaining inflammatory state follows. At tissue level the disease brings epithelial thickening with foci of erosion, deepened crypts loaded with detritus, fewer lymphoid follicles and germinal centres, capsular thickening and progressive stromal fibrosis (17). At cellular level the same process appears as dysregulated proliferation, persistent macrophage activation and disturbed apoptotic control, reflected in altered expression of Ki-67, CD68 and BCL-2. What is still missing is an integrated quantitative account that ties morphometric change, immunohistochemical reorganisation and clinical severity together.

Management remains contested despite advances in surgical technique (23). Tonsillectomy resolves severe disease but carries operative morbidity (24), removes a secondary lymphoid organ and is now performed under stricter indications; elective procedures have fallen in many health systems while emergency admissions have risen (5, 25). Interest in organ-preserving conservative protocols has grown accordingly, particularly where access to specialised surgical care is uneven. Uzbekistan is one such setting: a population above 35 million, most of it rural, and a post-Soviet health system still adapting its regional infrastructure to modern requirements (9). Ozone therapy has emerged here as a practical adjunct. Medical ozone has documented antimicrobial, anti-biofilm, anti-inflammatory and microcirculatory effects (10, 11), and pairing it with low-frequency ultrasound is intended to carry it deeper into the cryptal compartment while helping to clear pathological lacunar content.

Individual pieces of this picture have been studied separately, but morphometric, immunohistochemical and clinical outcomes have rarely been analysed together in a single cohort. Whether the structural and immunophenotypic changes of chronic tonsillitis move together under conservative therapy, and whether that movement can be measured objectively in tissue, has not been addressed systematically. We therefore set out to characterise the morphometric and immunohistochemical reorganisation of the palatine tonsil in chronic tonsillitis across age groups, and to evaluate the structural and clinical response to a standardised combined ozone-ultrasound protocol.

## Materials and methods

### Study design, participants and ethics

We carried out this single-centre clinical and morphological study at Bukhara State Medical Institute, Republic of Uzbekistan, with histological and immunohistochemical processing at the Regional Pathology Bureau of the Bukhara region. The institutional Expert Council approved the protocol (No. 23-Z/080), and the study followed the Declaration of Helsinki. Adult participants gave written informed consent; for children we obtained written consent from the legal guardians and age-appropriate verbal assent from the child. The work formed part of the institutional research programme on the upper respiratory tract in the post-COVID-19 period (Project No. 05.2022.DSc.174).

The morphological stage rested on 480 palatine tonsil specimens: histologically normal control tonsils (*n* = 160; 71 male, 89 female), chronic tonsillitis (*n* = 180; 90 male, 90 female) and specimens taken after a completed course of combined ozone-ultrasound therapy (*n* = 140; 60 male, 80 female). The clinical stage comprised 180 patients with chronic tonsillitis aged 4–40 years (101 male, 79 female). All of them received the ozone-ultrasound protocol and were assessed before and after the course. No parallel group receiving conventional conservative treatment alone was followed up for outcome comparison.

Control tissue came from planned surgery in patients without chronic tonsillitis, operated on for cysts and other benign non-inflammatory oropharyngeal lesions, and entered the control group only where clinical and histological signs of tonsillar inflammation were absent. It is a conditional age norm rather than intact tissue, because oropharyngeal lymphoid tissue meets environmental antigens continuously; the tissue inflammatory index in controls was 2.49 (SE 0.11) points on a 0–7 scale. Chronic tonsillitis specimens came from planned tonsillectomy on clinical indications. Post-treatment specimens came from tonsillectomy in patients of the clinical cohort whose surgical indications persisted after the course, subjective symptoms having regressed while the clinical form of the disease stayed unchanged; those who responded adequately kept their tonsils and did not enter the morphological stage. Of the 180 clinical participants, 140 (77.8%) therefore also contributed morphological material. The control and untreated chronic tonsillitis groups took part in the morphological stage only, and the fact that the latter also numbers 180 is coincidental. In all, 520 unique participants provided 660 observations. We did not record the numbers screened and excluded before enrolment. Supplementary Figure S1 sets out the flow.

Inclusion criteria were age 4–40 years, a diagnosis of chronic tonsillitis and written informed consent. The diagnosis rested on history, otorhinolaryngological examination, pharyngoscopy, palpation of the regional lymph nodes and endoscopy of the oropharynx, and required recurrent sore-throat episodes together with objective pharyngoscopic signs of chronic inflammation. Episode frequency was recorded categorically as absent, rare (up to three per year) or frequent (four or more per year); we did not use Paradise criteria for selection. Exclusions were acute infectious or oncological disease, decompensated cardiovascular, respiratory or endocrine disease, systemic autoimmune disease, acute purulent ENT inflammation, previous tonsillectomy or adenoidectomy, contraindications to surgery or ozone therapy, and refusal to participate.

### Clinical assessment

Subjective symptoms were scored individually: frequency of sore throat (0 = no complaints; 1 = rare episodes, up to three per year; 2 = frequent exacerbations, four or more per year), pain on swallowing (0 = absent, 1 = present), cryptal detritus (0 = no lacunar content; 1 = moderate accumulation in individual crypts; 2 = pronounced and widespread filling of the lacunae), halitosis (0 = absent, 1 = present) and regional lymph-node enlargement (0 = not palpable, 1 = palpable). Objective findings were quantified with the Tonsillitis Clinical Index, a scale of our own design. Five signs are each graded as absent (0), moderate (1) or pronounced (2): hyperaemia of the palatine arches, bead-like thickening of the arch margins, cicatricial adhesions between the tonsils and the arches, pathological lacunar content in the form of caseous masses or liquid pus, and regional lymphadenopathy. The total runs from 0 to 10 points, and bands of 0–3, 4–6 and 7–10 defined mild, moderate and severe disease. All measures were recorded before and after the course.

### Histopathology and immunohistochemistry

Specimens were fixed in 10% neutral buffered formalin for 24 h, processed through graded ethanol and xylene, embedded in paraffin, cut at 3–4 um on a rotary microtome and mounted on adhesive-coated slides. Routine sections were stained with haematoxylin and eosin, and stromal fibrosis was assessed additionally by Masson's trichrome. For immunohistochemistry we stained 3 um sections on Ventana adhesive slides using a Ventana BenchMark XT platform (Roche, Switzerland) with antibodies against Ki-67, CD68 and BCL-2 (Ventana, Switzerland; 1:100), visualised with DAB chromogen and counterstained with Mayer's haematoxylin. Positive and negative tissue controls ran in parallel in every batch.

Slides were examined on a Soptop CX40 light microscope (Soptop, China) with an OD400UHW10 four-megapixel imaging system and built-in morphometric software, at x100, x200 and x400, in at least ten high-power fields per specimen. Parameters were epithelial thickness (um), crypt depth (um), cryptal detritus score (0–3), epithelial erosion (%), lymphoid follicle density (n/mm2) and diameter (um), germinal centre count (n/mm2) and area (um2), capsular thickness (um), fibrotic stromal change (%) and a composite tissue inflammatory index (0–7 points).

Immunohistochemical expression was quantified by counting immunopositive cells in 5–10 randomly selected high-power fields per specimen at x400, expressed as cell density (cells/mm2) for Ki-67 and CD68 and as the proportion of positive cells (%) for BCL-2. Counts were recorded separately for the germinal centre, the mantle zone, the interfollicular zone and, for CD68, the cryptal region, together with subcellular localisation and staining intensity. Immunohistochemistry covered every specimen of the morphological stage.

### Combined ozone-ultrasound therapy

The protocol, registered as utility-model patent FAP20230057 of the Republic of Uzbekistan, was applied in addition to standard conservative treatment, which every patient in the clinical cohort received. Ozonated 0.9% saline at 4–6 mg/L (optimally about 5 mg/L, 35 +/- 1 degrees C) was generated with an MOP0.4-AD generator (AQUAPURE) running on medical oxygen and delivered into the lacunar lumen with simultaneous aspiration and synchronous low-frequency ultrasound (18–22 kHz; 0.4–0.7 W/cm2; 30–45 s per tonsil). Anti-biofilm treatment of the lacunae with N-acetylcysteine (1%–3%) followed, then adsorption-rheological treatment with polyvinylpyrrolidone (0.5%–3.0% w/w), controlled microdrying of the tonsillar surface, application of a prolonged-release ozone gel, and finally a mucoadhesive protective layer based on chitosan or hyaluronic acid. Courses ran to 3–7 sessions at intervals of one to two days: 3–4 sessions in mild disease, five in moderate and 6–7 in severe. Sessions were performed without anaesthesia, with the same regimen in children and adults. Tolerability was monitored clinically at every session by the treating otorhinolaryngologist, who recorded any mucosal injury, bleeding, laryngospasm, gag reflex severe enough to interrupt the procedure, or systemic reaction to ozone. No structured pain scale was applied.

### Statistical analysis

Analyses were run in Microsoft Excel and SPSS. Descriptive statistics included the mean, standard error, standard deviation and range, and normality was checked with the Shapiro–Wilk test. Morphometric parameters were compared between control and chronic tonsillitis by Student's t-test or the Mann–Whitney U-test. Age effects were examined by one-way analysis of variance within each group separately, and sex differences within age periods with Bonferroni correction (alpha = 0.0042). Differences in immunohistochemical expression across the three groups were assessed by one-way analysis of variance with pairwise *post-hoc* comparison, and associations between markers by Pearson's correlation coefficient. Paired pre- and post-treatment clinical scores were compared with the Wilcoxon signed-rank test, and we report 95% confidence intervals for the clinical outcomes. No *a priori* power calculation was performed; sample size followed from the material available during the study period. A *post-hoc* sensitivity analysis showed that at a two-sided alpha of 0.05% and 80% power the group sizes permit detection of a standardised difference of about d = 0.30 for the morphological comparisons and dz = 0.21 for the paired clinical comparison. Significance was set at *p* < 0.05.

## Results

### Participants and specimens

The morphological stage drew on 480 palatine tonsil specimens: histologically normal control tissue (*n* = 160), chronic tonsillitis (*n* = 180) and specimens obtained after combined ozone-ultrasound therapy (*n* = 140). The clinical stage comprised 180 patients aged 4–40 years, spread across six age periods and examined before and after the treatment course. Within the chronic tonsillitis group, severity was graded before surgery as mild in 26 patients (14.4%), moderate in 105 (58.3%) and severe in 49 (27.2%). Men and women were represented in comparable proportions in every group, and no morphometric parameter differed significantly by sex within any age period after Bonferroni correction.

### Morphometric reorganisation in chronic tonsillitis

Chronic tonsillitis reorganised the palatine tonsil in a consistent way (Table 1). The heaviest changes fell on the cryptal apparatus and the capsule rather than on the epithelial lining: crypt depth increased more than fivefold (+412.3%) and capsular thickness more than threefold (+252.6%), while epithelial thickness rose only marginally (+6.6%). Where the epithelium was involved, it broke down rather than thickened. Surface erosion covered 2.5 times more area (+152.5%) and the intracryptal detritus score rose by 53.1% (all *p* < 0.05).

**Table 1:** Morphometric parameters of the palatine tonsil in normal tissue and in chronic tonsillitis (mean and standard error).

Parameter	Normal tonsil (*n* = 160)	Chronic tonsillitis (*n* = 180)	Change, %
Epithelial thickness, um	120.05 +/- 0.79	127.92 +/- 1.57*	+6.6
Crypt depth, um	451.03 +/- 3.31	2310.80 +/- 21.94*	+412.3
Cryptal detritus score, points	1.44 +/- 0.09	2.21 +/- 0.03*	+53.1
Epithelial erosion, %	12.34 +/- 0.35	31.15 +/- 0.48*	+152.5
Lymphoid follicle density, n/mm2	6.51 +/- 0.06	2.95 +/- 0.06*	−54.7
Lymphoid follicle diameter, um	323.36 +/- 1.82	210.78 +/- 3.00*	−34.8
Germinal centre count, n/mm2	1.81 +/- 0.04	1.41 +/- 0.04*	−22.1
Germinal centre area, um2	41 420 +/- 427	39 634 +/- 962	−4.3 (NS)
Capsular thickness, um	94.89 +/- 0.90	334.55 +/- 3.02*	+252.6
Stromal fibrosis, %	18.04 +/- 0.41	27.78 +/- 0.50*	+54.0
Tissue inflammatory index, points	2.49 +/- 0.11	5.27 +/- 0.08*	+111.6

Values are pooled across six age periods and both sexes.

* *p* < 0.05 vs. normal tissue; NS, not significant.

The lymphoid compartment behaved differently. Follicle density fell by 54.7%, follicle diameter by 34.8% and the number of germinal centres by 22.1% (all *p* < 0.05). The area of the germinal centres that survived, however, barely moved: 41 420 (SE 427) against 39 634 (SE 962) um2, a difference of 4.3% that did not reach significance (*p* = 0.09). Lymphoid injury in chronic tonsillitis therefore works by thinning the follicular network rather than by shrinking individual germinal centres, whose proliferative capacity looks comparatively intact.

**Figure 1:**
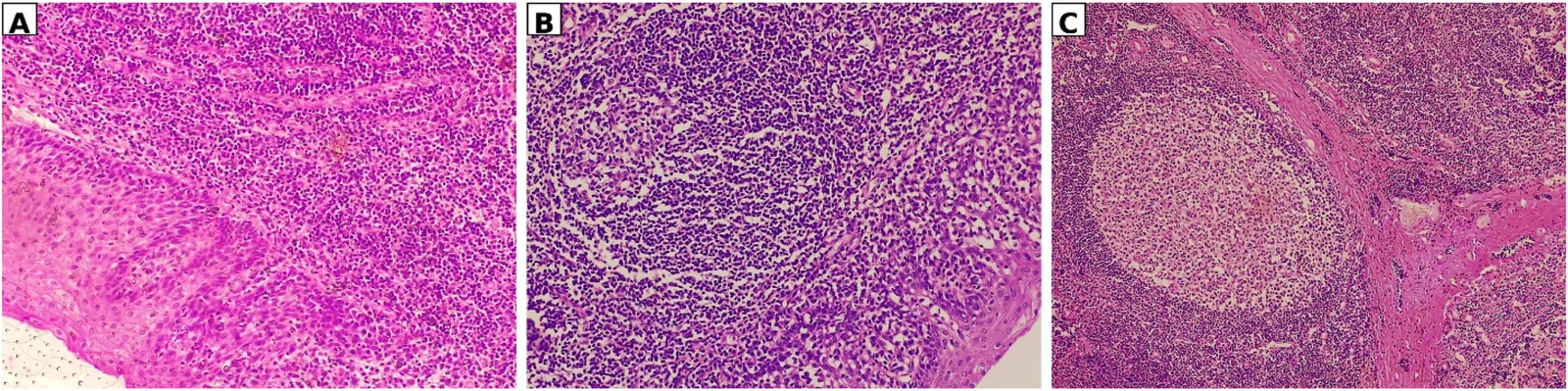
Representative haematoxylin and eosin sections of the palatine tonsil (original magnification: A and B x400; C x200). **(A)** Normal palatine tonsil from a child aged 4-7 years, with well-developed lymphoid tissue, moderately formed crypts and an intact multilayered squamous epithelium. **(B)** Normal palatine tonsil from an adult aged 36-40 years, with preserved follicular architecture, a clearly defined mantle zone and a thinned squamous epithelium, illustrating age-related involutional remodelling. **(C)** Chronic tonsillitis in a 35-year-old man, with lymphoid follicles showing well-preserved germinal centres, extensive hyalinosis of the interlobular crypts and hyalinised vessel walls in the stroma.

Stromal remodelling appeared as a 54.0% rise in the proportion of fibrotic tissue, and the composite tissue inflammatory index climbed from 2.49 (SE 0.11) to 5.27 (SE 0.08) points, an increase of 111.6% (*p* < 0.05). Age made no significant difference to any morphometric parameter in the control group (all *p* > 0.05). In chronic tonsillitis it did: epithelial thickness (F = 17.88; *p* < 0.001), erosion area (F = 17.56; *p* < 0.001), fibrotic fraction (F = 20.62; *p* < 0.001) and follicle diameter (F = 2.69; *p* = 0.023) all varied with age, which suggests that age-related remodelling of the palatine tonsil becomes visible only once chronic inflammation is present (Figure 1).

Stratifying by age showed that the alterations were not uniform across development (Supplementary Table S1). Crypt deepening, loss of follicle density, reduction in germinal centre number and capsular thickening were of almost identical magnitude in children and adolescents aged 4–16 and in adults aged 17–40. The epithelium behaved differently: thickness did not change in the paediatric stratum (−2.8%; *p* > 0.05) but rose in adults (+11.6%; *p* < 0.05), and erosion, stromal fibrosis and the tissue inflammatory index all increased further in adults. Germinal centre area was unchanged in both strata.

### Quantitative immunohistochemistry before and after therapy

**Figure 2:**
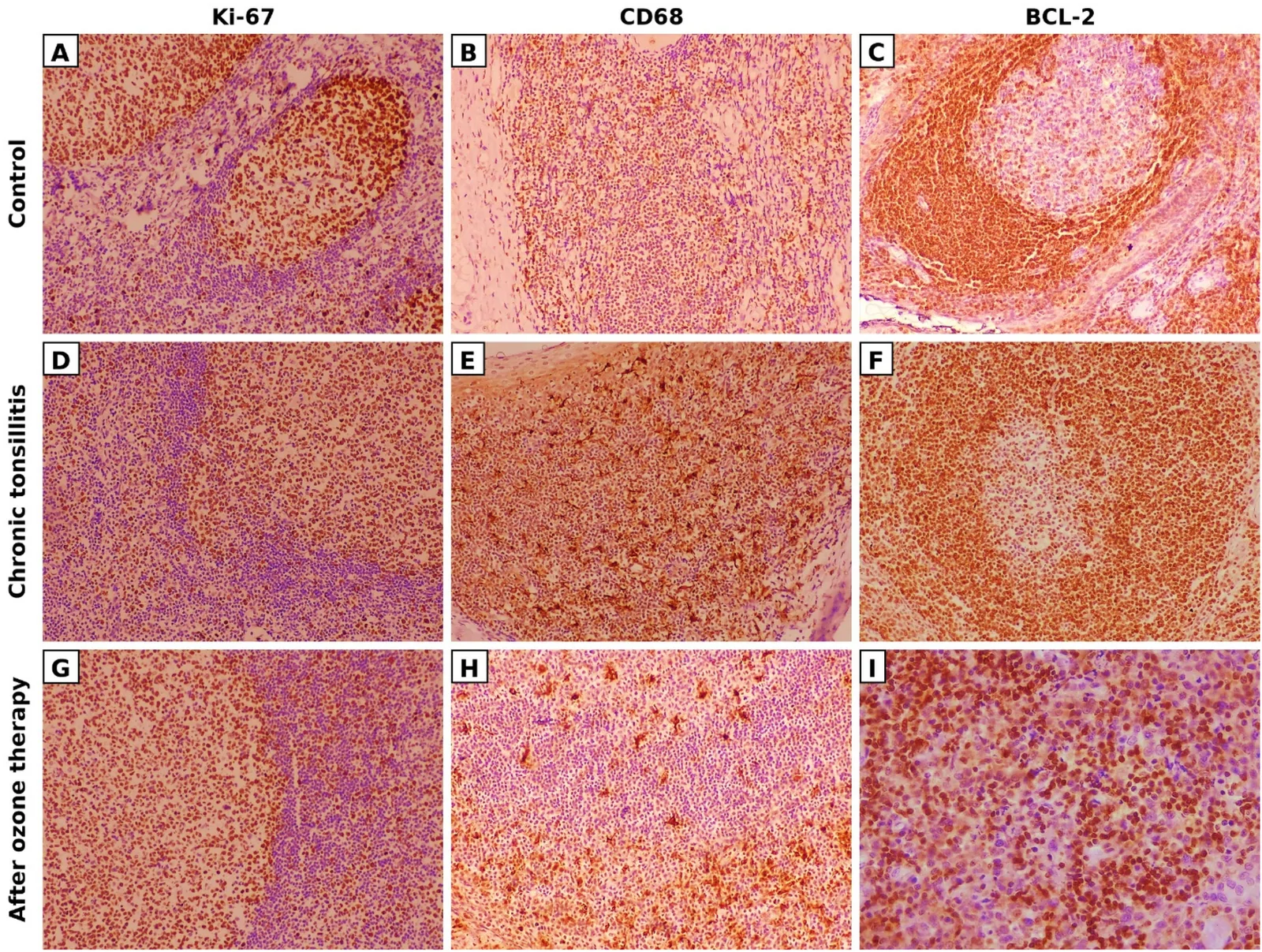
Immunohistochemical profile of the palatine tonsil for Ki-67, CD68 and BCL-2 across the three study groups (DAB chromogen, haematoxylin counterstain; original magnification: A and I x600; all other panels x400). **(A–C)** Control specimens: Ki-67 restricted to the germinal centres, tingible-body distribution of CD68, and zonal BCL-2 expression with mantle-zone positivity and negative germinal centres. **(D–F)** Chronic tonsillitis: diffuse Ki-67 positivity extending beyond the germinal centres, a stellate CD68 pattern, and pan-follicular BCL-2 expression with loss of zonality. **(G–I)** After combined ozone-ultrasound therapy: Ki-67 again restricted to the germinal centres, reduced CD68 expression with a moderate stellate pattern, and partial recovery of the zonal BCL-2 distribution. Quantitative data for all panels are given in Table 2.

Quantitative immunohistochemistry produced a clear three-stage pattern across the control, untreated and post-therapy groups (Table 2; Figure 2). In chronic tonsillitis, Ki-67 density rose in every compartment, and it rose most where proliferation is normally least: fourfold in the mantle zone and 2.8-fold in the interfollicular zone, against 1.9-fold inside the germinal centres. Proliferative activity had lost its usual topographic restriction. CD68-positive macrophage density increased 2.8- to 3.2-fold, with the steepest rise in the cryptal region, the compartment in direct contact with biofilm-protected microbial content. BCL-2 gave the most specific signal: expression inside the germinal centres, physiologically close to absent, climbed from 12% (SE 2) to 90% (SE 6), so the zonal organisation of apoptotic control had broken down (all *p* < 0.05 vs. control).

**Table 2:** Immunohistochemical expression of Ki-67, CD68 and BCL-2 by tissue compartment (mean and standard error).

Marker and compartment	Control (*n* = 160)	Chronic tonsillitis (*n* = 180)	After therapy (*n* = 140)
Ki-67, cells/mm2			
Germinal centre	8 420 +/- 530	15 840 +/- 1 240*	10 120 +/- 640^#^
Interfollicular zone	3 120 +/- 240	8 640 +/- 720*	4 260 +/- 350^#^
Mantle zone	1 180 +/- 110	4 720 +/- 430*	1 630 +/- 120^#^
CD68, cells/mm2			
Germinal centre	2 280 +/- 180	6 480 +/- 420*	3 380 +/- 260^#^
Interfollicular zone	2 460 +/- 210	7 120 +/- 530*	3 840 +/- 310^#^
Cryptal region	2 840 +/- 230	8 940 +/- 640*	4 620 +/- 350^#^
BCL-2, % positive cells			
Germinal centre	12 +/- 2	90 +/- 6*	35 +/- 3^#^
Mantle zone	55 +/- 4	92 +/- 5*	61 +/- 4^#^
Interfollicular zone	38 +/- 3	76 +/- 5*	44 +/- 3^#^

Immunopositive cells were counted in 5–10 randomly selected high-power fields per specimen at x400.

* *p* < 0.05 vs. control.

# *p* < 0.05 vs. chronic tonsillitis (one-way analysis of variance with pairwise *post-hoc* comparison).

After the treatment course all three markers moved back towards control values (all *p* < 0.05 vs. chronic tonsillitis). Ki-67 density fell by 36%–65% depending on compartment, CD68 density by 46%–48%, and germinal centre BCL-2 positivity from 90% (SE 6) to 35% (SE 3), with partial recovery of the zonal pattern, negative germinal centres and preserved mantle-zone positivity. The three markers were positively intercorrelated in chronic tonsillitis, most strongly Ki-67 with BCL-2 (r =  + 0.76), then Ki-67 with CD68 (r =  + 0.71) and CD68 with BCL-2 (r =  + 0.63); these associations weakened after therapy.

### Clinical and morphological outcomes

Treatment outcomes are reported as within-group changes from baseline in the 180 patients who received the ozone-ultrasound protocol. The conventionally treated group was not followed up as a parallel comparator and no between-group comparison was made, so what follows describes the response to the protocol and does not establish superiority over conventional conservative treatment.

Every clinical parameter improved after the course (Wilcoxon signed-rank test, all *p* < 0.001). The integrated tonsillitis clinical index fell from 4.28 (SE 0.14) to 2.78 (SE 0.09) points, a mean reduction of 1.50 points (95% CI 1.17–1.83), or 35.0%. Sore-throat frequency dropped from 0.96 (SE 0.06) to 0.63 (SE 0.04) points (mean change 0.33; 95% CI 0.19–0.47), pain on swallowing from 0.44 to 0.29 (0.15; 95% CI 0.06–0.24), cryptal detritus from 0.82 to 0.54 (0.28; 95% CI 0.14–0.42), halitosis from 0.41 to 0.27 (0.14; 95% CI 0.05–0.23) and cervical lymph-node enlargement from 0.36 to 0.24 (0.12; 95% CI 0.05–0.19). Confidence intervals for the mean change were derived without assuming a correlation between paired observations and are therefore conservative. Clinically, baseline severity was highest in children and adolescents, yet the relative reduction in the clinical index lay between 34.3% and 35.9% in all six age periods, so age did not modify the treatment effect (Supplementary Table S2).

Paired morphometry of the post-treatment specimens showed a matching structural response. Germinal centre number rose 1.64-fold and follicle density 1.33-fold, follicle diameter 1.21-fold and germinal centre area 1.30-fold, while capsular thickness fell 1.43-fold, cryptal detritus 1.17-fold and the tissue inflammatory index from 5.27 (SE 0.07) to 4.43 (SE 0.11) points, a 1.19-fold reduction (*p* < 0.001). The fibrotic fraction of the stroma did not change (*p* = 0.338). We recorded no procedure-related complications, no mucosal injury and no adverse reactions to ozone exposure.

## Discussion

Chronic tonsillitis is among the commonest forms of upper respiratory tract pathology. It runs a recurrent course, and objective tools for monitoring the effect of conservative treatment are still lacking (22). We approached that gap by combining quantitative morphometry with a three-marker immunohistochemical panel and applying both before and after a structured ozone-ultrasound protocol. The main finding is that the structural and immunophenotypic changes of chronic tonsillitis form a reproducible pattern, that this pattern can be measured objectively, and that it responds to therapy in a coordinated way.

Our morphometric data both confirm and refine earlier histopathological work. Crypt enlargement and detrital accumulation, described as the dominant morphological features of the disease (12, 21), were reproduced here, with crypt depth more than five times the control value and the detritus score up by 53.1%. The covering epithelium behaved less predictably. Instead of the hyperplastic thickening usually assumed, thickness rose only 6.6% while the eroded area grew 2.5-fold, so the epithelium met persistent inflammation mainly by breaking down. Perry noted the high regenerative capacity of the crypt epithelium (14); our material was dominated instead by regenerative exhaustion with persistent erosion, which fits a long-standing disease course. Tabou et al. described a different pattern in tonsillar hypertrophy, where follicular enlargement predominates (13).

The lymphoid findings were the most informative. Follicle density fell by more than half and germinal centre number by 22.1%, yet the area of the surviving germinal centres was essentially unchanged (−4.3%; *p* = 0.09). That dissociation matters. It indicates that lymphoid injury in chronic tonsillitis proceeds by reduction of the follicular network as a whole rather than by involution of individual germinal centres, whose proliferative capacity appears comparatively preserved. The observation qualifies the conventional reading of tonsillar lymphoid depletion, and it carries a practical edge: the number of germinal centres, not their size, was the parameter that responded most clearly to treatment. Vascular change alongside cellular infiltration is consistent with Brandtzaeg's account of the tonsillar microvasculature in immune traffic (15).

The age-stratified analysis separates two components of the lesion. Changes in the cryptal apparatus, the follicular network and the capsule were the same in children and in adults, which suggests they follow directly from persistent lacunar infection whatever the age of the host. Epithelial thickening and stromal fibrosis, by contrast, appeared only or mainly in adults, which fits a cumulative process requiring years of inflammation. The practical reading is that the cryptal and follicular changes are the more useful targets for monitoring conservative treatment in children, since they are already fully developed at that age.

The immunohistochemical results extend the earlier literature in two directions. First, quantification showed that the three markers capture complementary and compartment-specific aspects of the lesion. Ki-67 rose furthest outside the germinal centres, fourfold in the mantle zone against 1.9-fold within them, so proliferation lost its normal topography. CD68 rose most in the cryptal region, where tissue meets biofilm-protected microbial content. BCL-2 gave the sharpest signal of all, appearing inside germinal centres that are physiologically almost negative. Bant et al. argued for the diagnostic value of similar panels (16), and Mogoanta et al. documented pronounced immune activation in chronic tonsillitis specimens (17); our quantitative data agree with both. Second, the markers were positively intercorrelated and moved together under therapy, with the associations weakening afterwards. That supports using them jointly as a tissue-level surrogate of lesional activity, in line with the idea of systemic immunomorphological dysregulation (18, 20) and with the demonstration by Tabou et al. that immunohistochemical change affects all structural compartments at once (13).

The biological rationale for the observed effects fits established mechanisms of ozone action. Ozone therapy improves microcirculation, raises tissue oxygenation and has antiseptic, anti-inflammatory and immunomodulatory effects (10). Russo et al. reviewed its antibacterial, antiviral and biofilm-disrupting properties (11), which matter in the lacunar microenvironment, where biofilm-protected communities are a recognised cause of treatment failure. Our protocol targets the persistent infectious focus, impaired lacunar drainage and chronic immune dysregulation together. Ultrasound should carry ozone deeper into the crypts and help clear their content, while the mucoadhesive gel and film prolong mucosal contact and limit recolonisation (11). The convergent fall in the clinical severity index, the tissue inflammatory index and cryptal detritus, alongside recovery of the lymphoid compartment, gives indirect support to this multi-target mechanism. The fibrotic fraction, by contrast, did not change (*p* = 0.338), which indicates that established stromal sclerosis is not reversed and that the effect operates on the inflammatory and lymphoid components instead. No component of the protocol caused mucosal injury or local intolerance.

Several limitations deserve statement. The design is observational and single-centre, without random allocation, and the conventionally treated group was not followed up as a parallel comparator; comparative effectiveness against alternative regimens therefore cannot be established here, and the improvements we report are within-group changes rather than evidence of superiority. The composition of the post-treatment morphological group adds a second constraint. Tonsils were removed only where surgical indications persisted, so those specimens come from patients whose clinical response was incomplete, while patients who responded adequately kept their tonsils and could not contribute tissue. The tissue-level changes reported after therapy are therefore a conservative lower bound rather than an average effect. Immunohistochemistry was quantified by manual counting rather than whole-slide image analysis, and counting was not blinded. Procedural tolerability was assessed by clinical observation rather than with a validated pain scale, and pain scores were not collected separately by age group. No procedure-related complications were observed, but the absence of a structured instrument means that mild or transient discomfort, particularly in the youngest children, may have gone unrecorded. Smoking status, gastro-oesophageal reflux disease and allergic disease were not recorded and could not be adjusted for, although each may influence tonsillar histology independently of chronic inflammation; the clinical comparison is within-patient, which controls stable characteristics by design, but residual confounding of the morphological comparison cannot be excluded. The cohort was not selected using Paradise criteria and symptom duration was not recorded, so comparison with trials applying those criteria needs caution. We also did not record the numbers screened and excluded before enrolment, and follow-up covered short and medium-term outcomes only. Multicentre prospective work with longer follow-up is warranted, ideally alongside transcriptomic and microbiome profiling of tonsillar tissue, as recently demonstrated in related tonsil-associated inflammatory conditions (19).

Chronic tonsillitis is associated with a reproducible pattern of morphometric and immunohistochemical reorganisation of the palatine tonsil involving the epithelium, the lymphoid compartment and the stroma. Combined ozone-ultrasound therapy was accompanied by measurable normalisation of these parameters and by concordant clinical improvement, including a 1.54-fold fall in the clinical severity index and recovery of the lymphoid compartment, while established stromal fibrosis stayed unchanged. The integrated morphometric and immunohistochemical framework proposed here may serve as an objective tool for judging conservative treatment of chronic tonsillitis and could support individualised management of a common disease. Supplementary Figure S1. Flow of participants through the study. Of the 180 participants of the clinical stage, 140 underwent tonsillectomy after the treatment course because surgical indications persisted, and provided the post-treatment specimens; 40 kept their tonsils and contributed no tissue. The control and untreated chronic tonsillitis groups comprise separate individuals. The numbers screened and excluded before enrolment were not recorded.

## Data Availability

The raw data supporting the conclusions of this article will be made available by the authors, without undue reservation.
