# Supplementary material for: Morphometric and immunohistochemical reorganisation of the palatine tonsil in chronic tonsillitis and its response to combined ozone-ultrasound therapy
Source: Front Surg. 2026 Aug 26;13:1878051. doi: 10.3389/fsurg.2026.1878051 (PMC13553907; doi:10.3389/fsurg.2026.1878051)
Supplement: Supplementary file 1 [file Table1.docx]

**Supplementary Material**

*Morphometric and immunohistochemical reorganisation of the palatine tonsil in chronic tonsillitis and its response to combined ozone-ultrasound therapy*

Manuscript 1878051, Frontiers in Surgery, Otorhinolaryngology - Head and Neck Surgery

**Supplementary Figure S1.** Flow of participants through the study. Of the 180 participants of the clinical stage, 140 underwent tonsillectomy after the treatment course because surgical indications persisted, and provided the post-treatment specimens; 40 kept their tonsils and contributed no tissue. The control and untreated chronic tonsillitis groups comprise separate individuals who took part in the morphological stage only. In total, 520 unique participants provided 660 observations across the two stages. The numbers screened and excluded before enrolment were not recorded.

**Supplementary Table S1.** Morphometric parameters of the palatine tonsil in normal tissue and in chronic tonsillitis, stratified by age. Values are mean and standard error, pooled across both sexes within each stratum.

|  | **Children and adolescents, 4-16 years** | | | **Adults, 17-40 years** | | |
| --- | --- | --- | --- | --- | --- | --- |
| **Parameter** | **Normal (n = 56)** | **Chronic tonsillitis (n = 64)** | **Change, %** | **Normal (n = 104)** | **Chronic tonsillitis (n = 116)** | **Change, %** |
| Epithelial thickness, um | 117.66 +/- 1.19 | 114.42 +/- 2.61 | -2.8 (ns) | 121.33 +/- 1.02 | 135.37 +/- 1.59* | +11.6 |
| Crypt depth, um | 447.86 +/- 5.66 | 2281.09 +/- 36.50* | +409.3 | 452.74 +/- 4.10 | 2327.19 +/- 27.44* | +414.0 |
| Cryptal detritus score, points | 1.36 +/- 0.15 | 2.19 +/- 0.05* | +61.3 | 1.49 +/- 0.11 | 2.22 +/- 0.04* | +49.1 |
| Epithelial erosion, % | 12.62 +/- 0.59 | 28.07 +/- 0.83* | +122.5 | 12.19 +/- 0.44 | 32.85 +/- 0.52* | +169.5 |
| Lymphoid follicle density, n/mm2 | 6.44 +/- 0.11 | 2.97 +/- 0.09* | -53.9 | 6.55 +/- 0.08 | 2.94 +/- 0.08* | -55.1 |
| Lymphoid follicle diameter, um | 321.56 +/- 3.05 | 219.51 +/- 5.01* | -31.7 | 324.33 +/- 2.27 | 205.95 +/- 3.68* | -36.5 |
| Germinal centre count, n/mm2 | 1.81 +/- 0.06 | 1.40 +/- 0.07* | -22.6 | 1.82 +/- 0.04 | 1.42 +/- 0.05* | -21.9 |
| Germinal centre area, um2 | 41 598 +/- 693 | 39 299 +/- 1 586 | -5.5 (ns) | 41 324 +/- 544 | 39 820 +/- 1 214 | -3.6 (ns) |
| Capsular thickness, um | 95.41 +/- 1.52 | 335.05 +/- 4.32* | +251.2 | 94.61 +/- 1.12 | 334.28 +/- 4.05* | +253.3 |
| Stromal fibrosis, % | 16.86 +/- 0.68 | 24.28 +/- 0.91* | +44.0 | 18.67 +/- 0.50 | 29.71 +/- 0.52* | +59.1 |
| Tissue inflammatory index, points | 2.32 +/- 0.19 | 4.64 +/- 0.15* | +99.7 | 2.58 +/- 0.14 | 5.61 +/- 0.06* | +117.7 |

** p < 0.05 versus normal tissue within the same age stratum; ns, not significant. Crypt depth, follicle density, germinal centre count and capsular thickness change by almost identical amounts in the two strata. Epithelial thickness does not change in children but rises in adults, and erosion, stromal fibrosis and the tissue inflammatory index increase further in adults. Germinal centre area is unchanged in both strata.*

**Supplementary Table S2.** Tonsillitis Clinical Index before and after combined ozone-ultrasound therapy, by age period. Values are mean and standard error for the 180 patients of the clinical stage.

| **Age period, years** | **TCI before treatment** | **TCI after treatment** | **Reduction, %** |
| --- | --- | --- | --- |
| 4-7 | 4.86 +/- 0.32 | 3.17 +/- 0.21 | 34.8 |
| 8-12 | 4.29 +/- 0.30 | 2.79 +/- 0.20 | 35.0 |
| 13-16 | 4.99 +/- 0.40 | 3.20 +/- 0.26 | 35.9 |
| 17-21 | 3.79 +/- 0.52 | 2.49 +/- 0.34 | 34.3 |
| 22-35 | 4.03 +/- 0.26 | 2.62 +/- 0.17 | 35.0 |
| 36-40 | 3.48 +/- 0.42 | 2.27 +/- 0.27 | 34.8 |

*Baseline severity was highest in children and adolescents, but the relative reduction lay between 34.3% and 35.9% in every age period, so age did not modify the size of the treatment effect. All within-group comparisons were significant at p < 0.001 (Wilcoxon signed-rank test).*
